# Molecular Role of Carbohydrates in Acne and Seborrheic Dermatitis

**DOI:** 10.7759/cureus.116420

**Published:** 2026-09-17

**Authors:** Tiffany A Kumala, Thien Ly Nguyen, Khwaja Hasan, Alisha Kashyap, Rashid M Rashid

**Affiliations:** 1 Dermatology, University of Texas Health Science Center, Houston, USA; 2 Dermatology, Texas Tech University Health Sciences Center, El Paso, USA; 3 Pediatrics, University of Texas Southwestern Medical Center, Dallas, USA; 4 Dermatology, Mosaic Dermatology, Houston, USA

**Keywords:** acne vulgaris, carbohydrates, cutaneous inflammation, glycemic index, insulin–igf-1 signaling, malassezia, seborrheic dermatitis

## Abstract

Acne vulgaris and seborrheic dermatitis are common inflammatory disorders of sebaceous skin shaped by interactions among sebaceous activity, resident microorganisms, immune signaling, and host metabolism. This narrative review examines how high-glycemic dietary exposure may influence these disorders through insulin and insulin-like growth factor-1 (IGF-1) signaling, mammalian target of rapamycin complex 1 (mTORC1), sebaceous lipogenesis, and disease-specific microbial and inflammatory pathways. In acne, observational studies, mechanistic data, and small randomized trials support a biologically plausible model in which high glycemic index (GI) or glycemic load (GL) promotes hyperinsulinemia, IGF-1 signaling, PI3K/Akt/FoxO1/mTORC1 activation, altered sebaceous lipid production, follicular keratinization, and inflammation; however, clinical dietary trials remain inconsistent. Emerging observations with glucagon-like peptide-1 receptor agonists further suggest that systemic metabolic signaling may modify inflammatory follicular disease, although these data remain preliminary. In seborrheic dermatitis, *Malassezia*-host interactions, sebaceous lipids, barrier dysfunction, and inflammatory signaling are well established, but direct evidence linking dietary glycemic exposure to lesional IGF-1/mTORC1 activity or disease flares is lacking. The available evidence therefore supports dietary and metabolic factors as potential disease modifiers rather than uniform causes. Dietary counseling may be considered as an adjunct in selected patients with acne, whereas disease-specific carbohydrate restriction cannot currently be recommended for seborrheic dermatitis. Controlled mechanistic and interventional studies are needed to clarify causality and identify metabolically susceptible subgroups.

## Introduction and background

Acne vulgaris and seborrheic dermatitis are common inflammatory disorders of sebaceous skin characterized by interactions among cutaneous immunity, sebaceous gland activity, microbial communities, and host metabolic factors [1,2]. Although pharmacologic therapies remain the mainstay of treatment, diet has received increasing attention as a potentially modifiable contributor, particularly in acne [3].

Glycemic index (GI) describes how rapidly a carbohydrate-containing food raises blood glucose, whereas glycemic load (GL) also accounts for the amount of carbohydrate consumed. Rapid increases in blood glucose can stimulate insulin and related growth-factor signaling. These pathways are relevant to acne vulgaris and seborrheic dermatitis because both disorders involve sebaceous glands, which produce lipid-rich sebum that contributes to the follicular environment in acne and supports lipid-dependent *Malassezia* species in seborrheic dermatitis.

High-glycemic diets can induce hyperinsulinemia and alter insulin-like growth factor-1 (IGF-1) signaling, with downstream effects on keratinocyte proliferation, sebaceous gland function, and inflammatory responses [1,3]. Changes in the sebaceous microenvironment may also influence resident microbes, including Cutibacterium acnes in acne and *Malassezia* species in seborrheic dermatitis [4-6].

Although diet-skin interactions are increasingly studied, relatively little literature integrates carbohydrate-sensitive metabolic pathways across acne and seborrheic dermatitis while distinguishing their disease-specific microbial and inflammatory mechanisms. This review examines those shared and divergent pathways and their potential clinical implications.

## Review

Literature search and selection approach

This narrative review synthesized peer-reviewed literature addressing dietary glycemic exposure, insulin and insulin-like growth factor-1 (IGF-1) signaling, mammalian target of rapamycin complex 1 (mTORC1), sebaceous gland biology, *Cutibacterium acnes*, *Malassezia*, and diet-related clinical outcomes in acne vulgaris and seborrheic dermatitis. Targeted searches were conducted in PubMed and Google Scholar through September 2026 using combinations of terms including “acne”, “seborrheic dermatitis”, “carbohydrate”, “glycemic index”, “glycemic load”, “insulin”, “IGF-1”, “mTORC1”, “FoxO1”, “sebocyte”, “*Cutibacterium acnes*”, “*Malassezia*”, and “diet”. Reference lists of relevant reviews and clinical guidelines were also examined for additional studies. Peer-reviewed mechanistic studies, observational studies, randomized controlled trials, systematic reviews, meta-analyses, and clinical guidelines were included when they directly addressed the molecular mechanisms, clinical associations, or therapeutic implications relevant to the review question. Because this was a narrative rather than systematic review, study selection was not intended to be exhaustive, no formal risk-of-bias instrument was applied, and no Preferred Reporting Items for Systematic Reviews and Meta-Analyses (PRISMA)-based screening, meta-analysis, or other pooled quantitative synthesis was performed.

Overview of carbohydrates and cutaneous biology

Carbohydrate exposure is not biologically uniform. Simple sugars, refined starches, fiber-rich carbohydrates, and fructose-containing sweeteners differ in absorption and metabolic handling. GI and GL capture part of this variation but do not fully account for carbohydrate source or food matrix. Controlled studies comparing high-fructose corn syrup (HFCS) with sucrose have reported both distinct acute metabolic responses and broadly similar longer-term endocrine effects, arguing against treating all added sugars as metabolically interchangeable or assuming that HFCS is uniquely harmful [7,8]. Socioeconomic context may further modify exposure: lower socioeconomic status has been associated with greater sugar-sweetened beverage consumption in U.S. adults [9]. These relationships remain vulnerable to confounding by healthcare access, stress, total energy intake, and other dietary and lifestyle factors.

Insulin and IGF-1 signaling converge on nutrient-sensitive pathways, including mTORC1, that regulate cellular growth and lipid metabolism [1]. This axis can influence sebaceous activity, keratinocyte proliferation, and androgen bioavailability [1,10]. In a randomized trial of 66 adults with moderate to severe acne, two weeks of a low-GI/GL diet significantly reduced circulating IGF-1 concentrations [11].

Observational data in seborrheic dermatitis suggest dietary associations, but they do not establish a carbohydrate-specific causal pathway [12].

Molecular role of carbohydrates in acne vulgaris

High-GL dietary patterns are associated with acne in several observational studies [3,13]. Mechanistically, the IGF-1-induced PI3K/Akt/FoxO1/mTORC1 axis links nutrient signaling to sebocyte proliferation, lipogenesis, and keratinocyte differentiation [14,15].

Insulin and IGF-1 Signaling

Insulin and IGF-1 are key mediators between high-glycemic dietary exposure and acne-related sebaceous signaling [16,17]. High-GL meals can promote hyperinsulinemia and IGF-1 activity [16]. During puberty, physiologic insulin resistance and compensatory hyperinsulinemia may further amplify androgen and IGF-1 signaling; higher HOMA-IR values have been reported in some acne cohorts [18]. Both hormones amplify downstream signaling through insulin receptors, IGF-1 receptors, and fibroblast growth factor receptor-2b [16]. These hormones exhibit synergistic effects through their shared receptor pathways, augmenting mitogenic signaling that promotes sebocyte and keratinocyte proliferation [19]. Low-differentiated sebocytes show increased expression of insulin and IGF-1 receptors, resulting in production of acne-promoting sebum and inflammatory mediators [15].

IGF-1 also stimulates keratinocyte proliferation and follicular hyperkeratinization, contributing to comedone formation, which is a critical early step in acne pathogenesis [14,20]. Studies demonstrate that IGF-1 activates the IGF-1/IGF-1R system in the epidermis, inducing overexpression of Ki-67 (a proliferation marker) and filaggrin, while promoting altered keratinization of the pilosebaceous duct [14,20,21].

In addition to these effects, insulin and IGF-1 interact with androgen signaling through multiple mechanisms. The hormone stimulates 5α-reductase activity, adrenal and gonadal androgen synthesis, and androgen receptor signal transduction [16]. Importantly, hyperinsulinemia decreases sex hormone-binding globulin (SHBG) production, which increases bioavailable androgens that can act on sebaceous glands [1,10]. Patients with moderate/severe acne have significantly lower SHBG concentrations compared to those without acne [10]. High-GI dietary exposure may amplify androgenic signaling by lowering sex hormone-binding globulin, while milk can stimulate insulin and IGF-1 signaling [16,22]. These hormonal effects provide a plausible mechanism for the epidemiologic dairy-acne associations discussed below.

Sebum Production and Lipogenesis

mTORC1 activation promotes sebocyte proliferation and lipogenesis, linking insulin/IGF-1 signaling to sebum production [3,14]. Immunohistochemical studies demonstrate increased mTOR expression in acne lesions, including sebaceous glands [21]. Sterol regulatory element-binding protein-1 (SREBP-1) is a major lipogenic transcription factor downstream of mTORC1. IGF-1 increases SREBP-1 expression and sebocyte lipogenesis through PI3K/Akt signaling [23], and PI3K inhibition attenuates this lipogenic effect [19].

Increased sebum output can contribute to follicular occlusion and microcomedone formation [1]. Microcomedones are precursor lesions of clinical acne, but current models place hyperkeratinization within a broader interaction among dysseborrhea, *C. acnes *strain-level behavior, and early inflammation [4,24]. Low-differentiated sebocytes show decreased expression of PPARγ and increased levels of insulin and IGF-1 receptors, resulting in production of acne-promoting sebum and inflammatory mediators [15]. PPARγ is involved in both sebocyte differentiation and anti-inflammatory responses; its reduced expression in poorly differentiated sebocytes contributes to both altered sebum composition and increased inflammatory mediator production [25].

FoxO1 provides another nutrient-sensitive regulatory node. Acne lesions demonstrate increased cytoplasmic FoxO1 and mTOR expression [21]. Akt-mediated phosphorylation shifts FoxO1 from the nucleus to the cytoplasm, reducing its inhibitory effects on lipogenic and androgen-related signaling [14,21]. Loss of nuclear FoxO1 may thereby facilitate mTORC1/SREBP-1 activity and altered follicular homeostasis [26]. Pharmacologic modulation of this signaling network has also been described during isotretinoin therapy, further supporting the relevance of FoxO1/mTORC1 signaling in acne pathogenesis, although treatment-specific mechanisms are beyond the dietary focus of this review [26].

Inflammatory Pathways

Inflammation occurs early in acne and may precede overt follicular hyperkeratinization [24], arguing against a strictly occlusion-first model. Acne inflammation involves both innate and adaptive signaling. *C. acnes* can activate the NLRP3 inflammasome in monocytes and macrophages, promoting IL-1β release [27], while *C. acnes*-derived products stimulate IL-6 and IL-8 in sebocytes through TLR2/p38 MAPK pathways [28]. Other mediators include TNF-α, IFN-γ, IL-12, and matrix metalloproteinases [24,29]. Oxidative stress adds another layer: oxidized sebum lipids, including squalene, can promote keratinocyte proliferation and leukotriene B4-mediated inflammation, while reactive oxygen species generated during inflammasome activation can amplify cellular injury and signaling [24,27].

*C. acnes *also activates TLR2/TLR4 and other pattern-recognition pathways in sebocytes, keratinocytes, and macrophages [28-30]. Acne-associated microbial biology is increasingly framed around biofilm behavior, loss of phylotype diversity, enrichment of acne-associated phylotypes, and Th17-skewed inflammation rather than simple bacterial overgrowth [4]. These changes engage keratinocytes, monocytes/macrophages, CD4+ T cells, and Th1/Th17 populations [4,30].

Nutrient-sensitive signaling can further amplify this inflammatory network. mTORC1 promotes pro-inflammatory mediator production, while PI3K/Akt, MAPK, AMPK, and NF-κB pathways regulate inflammatory responses in sebocytes and infiltrating immune cells [3,14,15]. *C. acnes*-induced NF-κB/p38 MAPK signaling can influence lymphocytes, monocytes, macrophages, and neutrophils [14].

Sebocytes themselves function as immune regulators and contribute to cutaneous T-cell polarization toward a Th17 phenotype [31]. STAT3 may provide counter-regulatory control because its inhibition has been associated with enhanced *C. acnes*-induced IL-8 secretion [28].

Clinical Correlations

Observational studies generally support an association between acne and higher dietary glycemic exposure. Dietary GI and GL were significantly higher in one acne cohort [13], while the NutriNet-Santé prospective cohort associated fatty and sugary products, sugary beverages, milk, and an energy-dense dietary pattern with current adult acne after adjustment for measured confounders [3]. Dairy has also been extensively studied; two meta-analyses found positive associations between dairy or milk consumption and acne [32,33]. Because these studies are observational and heterogeneous, they support an epidemiologic association rather than causality or a universal recommendation for dietary restriction.

Randomized dietary trials provide more direct but inconsistent evidence. A 12-week Australian trial found greater reductions in acne lesion counts and improved insulin sensitivity with a low-GL diet [34], while a Korean randomized trial similarly reported clinical and histologic improvement [35]. A shorter two-week intervention reduced circulating IGF-1 but was not designed to establish a durable clinical effect [11]. Conversely, adding a low-GL diet to benzoyl peroxide did not significantly improve lesion counts in another trial [36]. A small study combining metformin with a hypocaloric diet also reported improvement in men with an altered metabolic profile and acne, although the independent effects of medication and diet could not be separated [37].

Current clinical guidance reflects these mixed findings. The 2024 American Academy of Dermatology guideline concluded that evidence was insufficient to recommend low-GI/GL diets, dairy or whey restriction, omega-3 fatty acids, or chocolate avoidance as acne treatments [38]. Dietary history and counseling may nevertheless be individualized and framed as adjunctive rather than as a substitute for evidence-based therapy.

Metabolic Modulation Beyond Diet

Emerging evidence from glucagon-like peptide-1 receptor agonists (GLP-1 RAs) provides preliminary support for a relationship between systemic metabolic signaling and inflammatory follicular disease. In a prospective single-arm study, semaglutide treatment was associated with reductions in acne severity, hidradenitis suppurativa activity, and sebaceous activity alongside metabolic improvement [39]. GLP-1 RAs have also been proposed as adjunctive therapy in hidradenitis suppurativa, particularly in patients with obesity or metabolic dysfunction [40]. These findings are hypothesis-generating; controlled studies are needed to determine how much of the dermatologic effect reflects weight loss, improved insulin sensitivity, direct anti-inflammatory actions, or other mechanisms.

Molecular role of carbohydrates in seborrheic dermatitis

Proposed Metabolic and Sebaceous Signaling

Direct evidence linking dietary glycemic exposure to seborrheic dermatitis is limited. A parallel pathway is biologically plausible because high-GI/GL intake can increase insulin/IGF-1 signaling and sebaceous lipogenesis, potentially altering the lipid environment in which *Malassezia* resides. One study reported higher serum IGF-1 levels in affected patients, but lesional mTORC1 activation and a causal dietary pathway have not been established [41].

Malassezia Metabolism

*Malassezia* species are lipid-dependent members of the normal skin microbiome and primarily use lipids derived from sebum [42]. *Malassezia* lipases hydrolyze triglycerides, releasing free fatty acids that can influence local irritation, inflammation, and barrier homeostasis [43]. Direct studies connecting dietary carbohydrate intake with *Malassezia* proliferation or seborrheic dermatitis activity are lacking [44].

Sebaceous lipids and local pH may also influence *Malassezia* enzyme activity. The *Malassezia restricta* lipase MrLip5 demonstrates pH-dependent expression and activity, with greater activity under more alkaline experimental conditions [43]. Whether this mechanism materially contributes to seborrheic dermatitis in vivo remains uncertain.

Skin Barrier Dysfunction and Inflammatory Signaling

Barrier dysfunction and altered host responses to *Malassezia* are central features of seborrheic dermatitis. *Malassezia*-derived lipids and metabolites can interact with keratinocytes and immune pathways, contributing to inflammation in susceptible skin [6,45].

*Malassezia*-derived free fatty acids can engage host pattern-recognition receptors, including TLR2, C-type lectin receptors, and NOD-like receptors, promoting NLRP3 inflammasome activation and IL-1β signaling [45]. γδ T-cell/IL-17 and mast-cell pathways have also been implicated, supporting a model of dysregulated host inflammation rather than simple failure to eliminate the organism [45].

Barrier disruption and increased transepidermal water loss (TEWL) are characteristic of seborrheic dermatitis. Clinical improvement with antifungal therapy supports an important role for *Malassezia*, but its presence alone is insufficient to explain disease; host susceptibility and inflammatory responses to fungal metabolites appear critical [45,46].

Clinical Correlations

The best available dietary evidence in seborrheic dermatitis is observational. In a cross-sectional analysis from the Rotterdam Study, a fruit-rich dietary pattern was associated with lower odds of seborrheic dermatitis, whereas a Western dietary pattern was associated with higher odds only in women [12]. These findings do not isolate GL as the causal exposure and cannot establish that dietary modification prevents or treats disease.

Reviews of diet and seborrheic dermatitis emphasize that evidence for specific nutritional interventions remains sparse and heterogeneous [44,47]. Nutrient deficiencies can produce seborrheic-like eruptions in selected settings, but this is distinct from evidence that supplementation or carbohydrate restriction treats routine seborrheic dermatitis.

Shared and divergent pathways between acne and seborrheic dermatitis

Acne vulgaris and seborrheic dermatitis are clinically distinct, but both involve sebaceous gland activity, cutaneous inflammation, and interactions with resident microorganisms. Their overlap is best viewed as a shared sebaceous-metabolic context rather than evidence of an identical dietary mechanism.

Interindividual susceptibility to metabolic signaling

Metabolic exposure does not produce a uniform cutaneous phenotype. Acanthosis nigricans and acrochordons are recognized cutaneous markers associated with insulin resistance, yet these findings can occur without acne and many patients with acne have no overt metabolic manifestations [48]. This discordance supports viewing insulin/IGF-1 signaling as a disease modifier rather than a sufficient cause. Genetic susceptibility may further segment clinical responses: a genome-wide association meta-analysis of more than 20,000 acne cases identified numerous susceptibility loci, reinforcing a polygenic contribution to disease risk [49]. Hormonal milieu, sebaceous responsiveness, microbial composition, and local immune signaling may therefore determine which phenotype emerges from a similar systemic metabolic environment.

Both diseases involve sebaceous lipids, but their downstream biology differs. In acne, metabolic signaling intersects with dysseborrhea, follicular hyperkeratinization, *C. acnes* strain-level behavior, and early inflammation [4,24,50], whereas seborrheic dermatitis is more strongly characterized by *Malassezia*-host interactions, barrier dysfunction, and inflammatory responses to fungal metabolites [5,45]. These distinctions help explain why similar metabolic exposures may produce different cutaneous phenotypes.

Figure 1 shows the shared and divergent carbohydrate-driven molecular pathways in acne vulgaris and seborrheic dermatitis. Table 1 presents a summary comparing the pathogenesis and effects of diet on acne vulgaris and seborrheic dermatitis.

**Figure 1:**
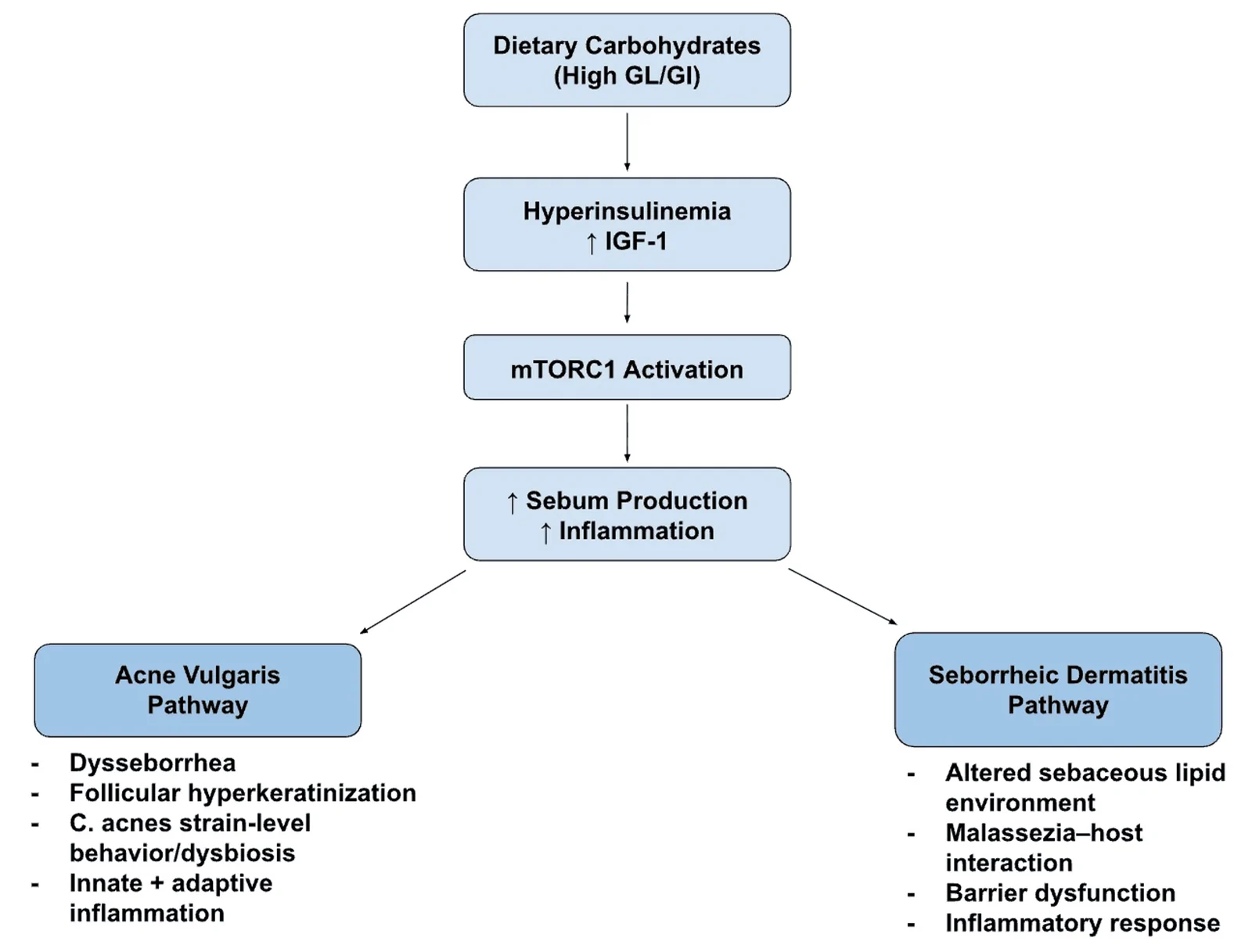
Shared and divergent carbohydrate-driven molecular pathways in acne vulgaris and seborrheic dermatitis. Schematic created by the authors based on published evidence regarding insulin/IGF-1/FoxO1/mTORC1 signaling, sebaceous gland biology, acne-associated inflammation and* C. acnes* interactions, and *Malassezia*–host interactions in seborrheic dermatitis [4–6,14,21,24,31,41,42,45,50].

**Table 1:** Summary comparing the pathogenesis and effects of diet on acne vulgaris and seborrheic dermatitis. The comparison was synthesized from published literature describing acne pathogenesis, insulin/IGF-1 and mTORC1 signaling, sebaceous gland and immune biology, *Cutibacterium acnes*, and *Malassezia*-host interactions, dietary intervention studies, and clinical evidence regarding diet in both disorders [1,4-6,14,21,24,30,31,34-38,41,42,45,47].

Feature	Acne vulgaris	Seborrheic dermatitis
Primary pathogenic context	Dysseborrhea + follicular hyperkeratinization + C. acnes dysbiosis + early inflammation	*Malassezia*–host interaction + sebaceous lipid environment + barrier dysfunction
Key metabolic pathway	Insulin-IGF-1 → mTORC1 activation	Proposed insulin/IGF-1–sebum link; lesional mTORC1 unproven
Sebum effects	↑ Sebum + altered composition → comedogenesis	↑ Sebum → lipid-rich environment for fungi
Host-microbe interaction	C. acnes strain-level dysbiosis and exaggerated follicular immune response	Altered host response to commensal *Malassezia* in a lipid-rich, barrier-impaired environment
Inflammation	IL-1β, TNF-α, Th17 pathways	IL-17, NLRP3 inflammasome, mast cell activation
Barrier function	Secondary disruption	Primary feature (↑ TEWL, dysfunction)
Immune cell response	Monocytes/macrophages (TLR2/NLRP3 → IL-1β), CD4+/Th1/Th17 cells; neutrophil recruitment contributes to pustular inflammation	Keratinocyte innate signaling, Th17/IL-17 responses, NLRP3 inflammasome activity, dendritic/mast-cell responses; inflammatory recruitment in response to *Malassezia* metabolites
Clinical evidence for diet	Moderate (RCT + cohort support)	Limited (mostly observational/indirect)

Clinical implications

Pharmacologic therapy remains the cornerstone of treatment for both acne vulgaris and seborrheic dermatitis. In acne, low-GI/GL dietary patterns may be considered as a complementary strategy for interested patients, particularly when incorporated alongside established topical or systemic therapy. Although current evidence is insufficient to support diet as a stand-alone treatment, the observed links between glycemic exposure, insulin/IGF-1 signaling, and acne severity suggest that dietary modification may have a meaningful adjunctive role in selected patients [38].

For seborrheic dermatitis, the evidence remains more preliminary. Observational dietary associations and the lipid dependence of *Malassezia* are hypothesis-generating rather than treatment-defining, and disease-specific carbohydrate restriction cannot currently be recommended [12,47]. However, these findings provide a rationale for future studies evaluating whether metabolic or dietary interventions can modify sebaceous lipid production, host-microbe interactions, or inflammatory responses in susceptible patients.

Dietary counseling is therefore best individualized and framed as an adjunct to evidence-based therapy. As mechanistic and clinical evidence evolves, metabolic and nutritional interventions may ultimately become a more defined component of multimodal management for acne and, potentially, seborrheic dermatitis.

Limitations of current evidence

The current literature is limited by the predominance of observational studies, variable dietary assessment methods, self-reported intake, residual confounding, and heterogeneous populations and outcome measures. Acne intervention trials are generally small and differ in duration, weight change, background therapy, and dietary composition. The evidence gap is larger in seborrheic dermatitis, where most proposed metabolic links remain indirect.

Future directions

Future studies should separate GL from total energy intake and weight change, standardize background therapy and clinical outcomes, and incorporate mechanistic biomarkers such as insulin, IGF-1, mTORC1 activity, sebum composition, and microbial behavior. In seborrheic dermatitis, prospective studies should first establish whether glycemic exposure is associated with disease activity before testing mechanistic or therapeutic pathways. Similar disease-specific frameworks may also be useful in other inflammatory dermatoses characterized by metabolic, barrier, and immune interactions.

## Conclusions

High-glycemic dietary exposure has a biologically plausible relationship with acne through insulin/IGF-1 signaling, mTORC1 activity, sebaceous lipogenesis, follicular keratinization, and inflammation, although clinical dietary trials remain mixed. In seborrheic dermatitis, *Malassezia*-host interactions, sebaceous lipids, and barrier dysfunction are established, but a direct carbohydrate-sensitive mTORC1 pathway remains unproven. The available evidence therefore supports a model in which metabolic exposures modify disease risk in susceptible hosts rather than produce a uniform cutaneous response. Further controlled and mechanistic studies are needed before disease-specific dietary recommendations can be made.
